# Reductions in HIV/STI Incidence and Sharing of Injection Equipment among Female Sex Workers Who Inject Drugs: Results from a Randomized Controlled Trial

**DOI:** 10.1371/journal.pone.0065812

**Published:** 2013-06-13

**Authors:** Steffanie A. Strathdee, Daniela Abramovitz, Remedios Lozada, Gustavo Martinez, Maria Gudelia Rangel, Alicia Vera, Hugo Staines, Carlos Magis-Rodriguez, Thomas L. Patterson

**Affiliations:** 1 University of California San Diego, Department of Medicine, La Jolla, California, United States of America; 2 Instituto de Servicios de Salud Publica, Tijuana, Baja California, Mexico; 3 Federacion Mexicana de Asociaciones Privadas, Ciudad Juarez, Chihuahua, Mexico; 4 Colegio de la Frontera Norte, Tijuana, Baja California, Mexico; 5 Universidad Autonoma de Ciudad Juarez, Ciudad Juarez, Chihuahua, Mexico; 6 Concorcio de Investigacion sobre HIV/AIDS and TB, Mexico City, Mexico; 7 University of California San Diego, Department of Psychiatry, La Jolla, California, United States of America; UNAIDS, Switzerland

## Abstract

**Background:**

We evaluated brief combination interventions to simultaneously reduce sexual and injection risks among female sex workers who inject drugs (FSW-IDUs) in Tijuana and Ciudad Juarez, Mexico during 2008–2010, when harm reduction coverage was expanding rapidly in Tijuana, but less so in Juarez.

**Methods:**

FSW-IDUs ≥18 years reporting sharing injection equipment and unprotected sex with clients within the last month participated in a randomized factorial trial comparing four brief, single-session conditions combining either an interactive or didactic version of a sexual risk intervention to promote safer sex in the context of drug use, and an injection risk intervention to reduce sharing of needles/injection paraphernalia. Women underwent quarterly interviews and testing for HIV, syphilis, gonorrhea, *Chlamydia* and *Trichomonas,* blinding interviewers and assessors to assignment. Poisson regression with robust variance estimation and repeated measures ordinal logistic regression examined effects on combined HIV/STI incidence and receptive needle sharing frequency.

**Findings:**

Of 584 initially HIV-negative FSW-IDUs, retention was ≥90%. After 12 months, HIV/STI incidence decreased >50% in the interactive vs. didactic sex intervention (Tijuana:AdjRR:0.38,95% CI:0.16–0.89; Juarez: AdjRR:0.44,95% CI:0.19–0.99). In Juarez, women receiving interactive vs. didactic injection risk interventions decreased receptive needle-sharing by 85% vs. 71%, respectively (p = 0.04); in Tijuana, receptive needle sharing declined by 95%, but was similar in active versus didactic groups. Tijuana women reported significant increases in access to syringes and condoms, but Juarez women did not.

**Interpretation:**

After 12 months in both cities, the interactive sexual risk intervention significantly reduced HIV/STI incidence. Expanding free access to sterile syringes coupled with brief, didactic education on safer injection was necessary and sufficient for achieving robust, sustained injection risk reductions in Tijuana. In the absence of expanding syringe access in Juarez, the injection risk intervention achieved significant, albeit more modest reductions, suggesting that community-level interventions incorporating harm reduction are more powerful than individual-level interventions.

**Trial Registration:**

clinicaltrials.gov NCT00840658

## Introduction

Globally, female sex workers (FSWs) experience elevated risks of acquiring HIV and other sexually transmitted infections (STIs). In a recent meta-analysis of 50 countries, overall HIV prevalence was 11.8% and compared to other women of reproductive age, the pooled odds ratio of HIV infection among FSWs was 13.5% [1]. These estimates under-represent HIV infection among FSWs who inject drugs (FSW-IDUs) who experience heightened risk of HIV and STIs through two transmission routes: unprotected sexual intercourse and sharing injection equipment with intimate partners, clients and peers [2].

FSW-IDUs are vulnerable to acquiring and transmitting HIV/STIs because of biological, behavioural and structural factors [2]. Biologically, the probability of acquiring HIV per intravenous drug injection is between 0.63% to 2.4% [3], and the probability of acquiring HIV per unprotected male-to-female vaginal sex act is 0.124% [4]. However, the probability of a male acquiring HIV from an HIV-infected FSW is estimated at 2.442% per transaction [4].

Addiction can elevate the probability of sharing injection equipment or having unprotected sex. Engaging in sex work under the influence of drugs, or injecting drugs with clients [5] can compromise one’s ability to negotiate safe sex [6] or avoid sharing injection equipment. Drug use and accompanying withdrawal symptoms have also been associated with having unprotected sex in exchange for more money [7] or being more likely to acquiesce to clients’ demands to forgo condoms [8], [9]. Structural factors related to policing practices and working in outdoor spaces have also been associated with elevated HIV risks among FSWs who use injection and non-injection drugs [5], [10].

Considerable overlap between FSW and IDU populations has been documented in parts of Southeast and Central Asia [11], [12], Eastern Europe[13]–[15] Africa [16], Latin America [17] and North America [18], including Mexico [19]. In an earlier study of FSWs in Tijuana and Ciudad (Cd.) Juarez –two Mexican-U.S. border cities adjacent to San Diego, CA and El Paso, TX respectively– 18% of FSWs reported injecting drugs [8]. In Cd. Juarez and Tijuana, HIV prevalence among FSWs increased from 2% in the 1990’s to 8% by 2006 [20], and 12% among FSW-IDUs) [8]. In 2008, the states of Chihuahua and Baja California, where these cities are located ranked 3rd and 4th in HIV prevalence respectively among Mexico’s 32 states [21]. In Tijuana alone, approximately one in 112 adults aged 15–49 was HIV-infected [22]. HIV prevalence among IDUs in Tijuana has remained stable at 4% among males but was 10% among females in 2006–2007 [23]; in Cd. Juarez, HIV prevalence rose from 4% in 2005 [24] to 7% in 2011 (personal communication, Dr. Carlos Magis-Rodriguez, 2012).

Tijuana and Cd. Juarez are both located on major drug trafficking corridors through which heroin, methamphetamine and cocaine are transported [25]. In both cities, injection and non-injection stimulant use were both independently associated with HIV infection among FSWs [20], and prevalence of infectious syphilis (i.e., titers ≥1∶8), gonorrhea and Chlamydia was significantly higher among FSW-IDUs at 22.7%, 15.2% and 21.2% compared to 13.1%, 5.2% and 11.9%, respectively among other FSWs [8]. In Tijuana, half of female IDUs reported trading sex, among whom HIV prevalence was 10% [23].

Efforts to reduce HIV risk among the most vulnerable FSWs are needed since FSWs account for nearly one fifth of reported HIV infections among women of reproductive age in Mexico, and because the quasi-legal status of sex work in Mexico attracts large numbers of clients from the U.S. and elsewhere. Globally, there is a dearth of interventions that have focused on FSWs who use drugs [2].

We previously conducted a two-arm randomized trial *Mujer Segura* (Safe Woman) from 2004–2006 to evaluate whether a thirty-minute, theoretically-based motivational interviewing (MI) intervention would significantly increase negotiation of condom use among FSWs in Tijuana and Cd. Juarez. The intervention was associated with a 40% reduction in combined HIV/STI incidence, significantly fewer unprotected sex acts compared to the control condition [26] and was cost-effective [27]. However, FSW-IDUs improved less than FSWs who did not inject drugs and the frequency of needle sharing was unchanged, which was anticipated since this intervention did not attempt to intervene upon drug use behaviors or sexual risk behaviors in the context of drug use [28].

Recognizing that interventions which narrowly focus only on safer sex or safer injection will be of limited effectiveness for FSW-IDUs given their extreme vulnerability, we designed a four-arm factorial randomized control trial called *Mujer Mas Segura* (Safer Woman) to simultaneously test the efficacy of two behavioral interventions aimed at increasing condom use in the context of ongoing drug use and decreasing sharing of injection equipment among FSW-IDUs. Women received both interventions in one of two formats, an interactive version or a didactic version that served as a control. We hypothesized that the joint effects of the interactive format of both interventions would generate greater risk reductions compared to the didactic formats. We also examined whether intervention effects differed between the two cities since availability of sterile syringes, injection paraphernalia and condoms began expanding rapidly in Tijuana during the study period, but was more limited in Cd. Juarez [29].

## Methods

### Ethics Statement

Women deemed potentially eligible underwent written informed consent and were queried to ensure that they understood what was required for study participation. The study protocol was approved by Institutional Review Boards in the US (University of California, San Diego, [UCSD]), and Mexico (Centro Nacional para la Prevencion de VIH/SIDA [CENSIDA], Universidad Autonoma de Ciudad Juarez and Hospital General de Tijuana.

### Participants

Between October, 2008 and July, 2010, FSW-IDUs were recruited into a randomized controlled trial in Tijuana and Cd. Juarez, Mexico, as described previously [29] (see Checklist S1 and Protocol S1).

#### Inclusion criteria

Participants were required to: (i) be biologically female, (ii) be at least 18 years old, (iii) report exchanging sex for money, drugs, shelter or goods in the last month, (iv) report injecting drugs at least once in the last month, (v) report having had unprotected vaginal or anal sex with male clients at least once during the previous month and (vi) report having shared needles, syringes or other injection paraphernalia (i.e., cookers, cotton, rinse water) at least once within the last month; (vii) test HIV-negative at baseline, and (viii) agree to receive antibiotic treatment for Chlamydia, gonorrhea or syphilis.

#### Recruitment

As previously described, project staff approached women at venues frequented by FSWs and IDUs in both cities (e.g., motels, hotels, brothels, shooting galleries, bars, alleys, street corners). Women expressing interest in the study were referred to the project office or a mobile unit for eligibility screening. A five-minute survey served as a screener for study eligibility, based on the above criteria. Staff also checked for injection stigmata (i.e., track marks). Women deemed potentially eligible underwent informed consent and HIV rapid tests.

### Data Collection

#### Baseline interview

Surveys were administered using computer-assisted personal interviewing (CAPI; NOVA software, MD, USA), by bi-cultural female staff who were familiar with the local population. The baseline survey collected data on socio-demographic and family background, sources of income, history, practices and environmental influences regarding substance use (type and frequency of injection and non-injection drug use, alcohol use), receptive and distributive sharing of syringes, injection and non-injection paraphernalia, frequency of injection and syringe sharing, syringe cleaning, needing help injecting, drug use and needle sharing in jail and history of drug treatment. We also collected data on accessibility of sterile syringes, barriers to purchasing and carrying syringes, shooting gallery attendance, and frequency of arrest and incarceration for charges related to drug possession and paraphernalia. FSWs were also asked to report whether they used alcohol or used injection and non-injection drugs before or during sex with regular, casual and client partners over the past month.

Sexual behaviors included number and frequency of unprotected vaginal and anal sex with clients, spouse/steady partners and casual male partners in the past month; number of partners who inject drugs, number of female sex partners, and use of the male and/or female condom.

We also collected data on contextual factors such as work setting, having a pimp or manager, selected client characteristics (e.g., demands for unprotected sex, client aggression or violence), amounts received for protected vs. unprotected vaginal and anal sex, and perceived changes in HIV/STI prevention services (i.e., availability of condoms, sterile syringes, HIV testing and medical care). Follow-up interviews and HIV/STI testing were conducted four, eight and twelve months’ post-randomization.

#### Randomization

Participants were assigned to one of four groups based on a randomization schedule that was generated *a priori* by the study statistician that was not disclosed to the interviewers, ensuring that they were blind to group assignment.

#### Intervention and control conditions

584 HIV-negative women meeting our eligibility criteria (284 in Tijuana and 300 in Ciudad Juarez) were randomized to one of four conditions in a 2×2 factorial design. Each of the four conditions was delivered by trained female, bicultural counselors and included either an interactive or didactic version of the injection risk intervention and the sexual risk intervention, each 30 minutes in length. Therefore, each of the four conditions lasted approximately 60 minutes to complete, which served as an attention control. These modules were described in detail previously [29] and are summarized below.

Group A (i.e., *Didactic Injection Risk Intervention and Didactic Sexual Risk Intervention*) represented the control condition and took 60 minutes to complete. This group received information on both safer injection and safer sex that was delivered in a lecture-style format which was based on information in printed materials available at local health centers. Counselors were instructed not to encourage discussion. In the Didactic Injection Risk portion of the Intervention (30 minutes), the counselor stressed the importance of using sterile injection equipment to protect against HIV and viral hepatitis and the risks of transmission from sharing injection equipment, provided referrals to the local needle exchange and instructions on how to disinfect syringes with bleach when sterile syringes were unavailable. No theory-driven or skills-building elements focusing on safer injection were included. In the Didactic Sexual Risk portion of the Intervention (30 minutes), the counselor presented information based on a modified version of the CDC guidelines for HIV counseling, testing, and referral [30] and materials from CENSIDA that were used in the control condition of *Mujer Segura* [26]. No theory-driven active skills-building elements focused on safer sex were included.

For Group B (i.e. the Interactive Injection Risk Intervention and Didactic Sexual Risk Intervention), participants received the interactive injection risk reduction intervention and the didactic sexual risk intervention which also required 60 minutes to complete. The Interactive Injection Risk portion of the Intervention required approximately 30 minutes to complete. Components from the injection risk intervention were adapted from two randomized behavioral intervention trials conducted in the U.S. that were efficacious in reducing injection risks among IDUs [31] and incorporated Motivational Interviewing (MI), Social Cognitive Theory (SCT), and Theory of Reasoned Action (TRA). Both interventions significantly reduced receptive and distributive sharing of injection equipment [32], [33] (http://www.cdc.gov/hiv/topics/testing/non-healthcare/index.htm) [34]. Participants viewed a 4-minute video called “*Una Gota de Sangre*” that was created for the project and featured FSW-IDUs from Tijuana. The video depicted an improvised scene that illustrated how injection equipment can become contaminated with blood-borne viruses if injection equipment is shared, using a drop of fluorescent dye to simulate a drop of blood. When viewed under a black light, the cooker, cotton, water and even the fingers of the woman who prepared the injection equipment glowed in the dark, demonstrating how easily contamination of these items can occur. Women were also taught how to disinfect their syringes with bleach.

The counselor then used a ‘risk ladder’ to illustrate injection behaviors ordered from least risky (i.e. not using drugs) to most risky (i.e., using somebody’s syringe, etc.), and an action item for each that was written on a flash card. The participant was asked to place the card along the ladder, justifying its location. The counselor then used MI techniques to elicit information on the woman’s own risky injection behaviors, her perceived advantages and disadvantages of doing so, and the “decisional balance” approach to facilitate her personal realization that the negative outcomes associated with these behaviors outweighed the positive outcomes. The counselor then prompted the participant to verbally propose possible alternatives to sharing injection equipment, which helped build her personal motivation for change. A short role play was then used to help her identify barriers to safer injection, through which women practiced negotiating safer injection skills. Finally, participants were encouraged to set at least one goal to reduce their injection risks.

Group C, the Interactive Sexual Risk Intervention Condition and Didactic Injection Risk Intervention Condition, required 60 minutes to complete. Participants randomized to this condition and received the interactive sexual risk reduction intervention, and the didactic injection risk intervention described previously. The Interactive Sexual Risk portion of the Intervention required approximately 30 minutes to complete. The interactive sexual risk intervention was based on components of the *Mujer Segura* [26] and *Fastlane* [35] interventions, both of which combined the principles of SCT and TRA and used MI to facilitate condom negotiation skills. Components of these interventions were adapted to incorporate strategies for negotiating condom use within the context of their own, or their partner’s substance use, and were extensively piloted among FSW-IDUs in Tijuana and Cd. Juarez as previously described [29].

Briefly, the counselor and the participant discussed her awareness of unsafe sex and associated risks (e.g., HIV, STIs, pregnancy). The counselor probed the participant on her experiences with condom use/non-use, substance use during sex, and her perceived need and desire to change high risk sexual behaviors. She then showed the participant how to put a condom on properly using a model. The counselor then used MI techniques to prompt the participant to discuss the advantages and disadvantages she perceived to having unprotected sex and barriers to using condoms with regular or casual partners. The counselor then used the “decisional balance” approach to help the participant articulate that, in most cases, the positive consequences associated with condom use strongly outweighed the negative consequences. The counselor then helped the participant actively problem-solve her personal barriers to condom use, define achievable goals and arrive at a plan of action. The participant and counselor then engaged in a role-play to practice her condom negotiation skills.

Group D, the Interactive Injection Risk Intervention and Interactive Sexual Risk Intervention, also required 60 minutes to complete. Participants randomized to this condition received the interactive modules of the injection risk intervention and the sexual risk intervention, described above.

#### Outcome ascertainment

At baseline and quarterly for 12 months, all subjects were tested for HIV and four other STIs (*Treponema pallidum, Neisseria gonorrhoeae, Chlamydia trachomatis,* and *Trichomonas vaginalis)*. The primary intervention outcome was combined incidence of HIV/STIs, therefore those infected at baseline were excluded. HIV serostatus was ascertained using the “Determine”® rapid antibody test (Abbott Pharmaceuticals, Boston, MA); reactive samples were confirmed using an HIV-1 enzyme immunoassay and immunofluorescence assay. Syphilis serology was ascertained by the rapid plasma reagin (RPR) test (Determine™ Syphilis TP; Abbott Pharmaceuticals, Boston, MA); RPR-positive tests were subjected to the *Treponema pallidum* particle agglutination assay (TPPA) (Fujirebio, Wilmington, DE, USA). Syphilis titers ≥1∶8 were considered to be active infections. Initially, testing for Gonorrhoea and Chlamydia (GC/CT) was conducted using a vaginal swab rapid test kit (BioStar® OIA® GC and CHLAMYDIA) and positive samples were confirmed on urine specimens using Transcription-Mediated Amplification (TMA) (Genprobe, San Diego, CA). Following a change in CDC recommendations [36], the GC/CT protocol was modified in March 2009 to accommodate GC/CT urine screening and TMA on all specimens. *Trichomonas vaginalis* was detected from vaginal swabs using the OSOM® Trichomonas Rapid Test (Genzyme diagnostics, San Diego, CA). The San Diego County Health Department (SDCPHL) conducted all confirmatory tests.

#### Pre- and post-test counseling and referrals

Pre-test HIV/STI counseling was performed as per CDC and Mexican guidelines. Participants with an indeterminate or reactive HIV rapid test were referred to Municipal specialty clinic (CAPASITS) for further expedited follow-up while confirmatory test results were pending. Women testing positive for STIs were provided immediate free treatment by the study nurse. At quarterly follow-up visits, participants were re-tested for HIV and STIs and underwent follow-up interviews with recall periods that referred to the period since the last interview. Participants received modest monetary reimbursements ranging from $5 to $25 USD for baseline and follow-up visits, and ‘check-ins’.

### Statistical Analysis

To ensure that randomization assured balance between the intervention groups, we compared the four intervention groups with respect to binary outcomes by using Chi-Squared tests and with respect to continuous outcomes by using Kruskal-Wallis tests. Combined HIV/STI incidence density was calculated by taking the ratio between the number of incident cases and the number of person-years at risk accumulated over the 12-month study period for participants who had at least one follow-up visit and tested negative at baseline for HIV and any of the aforementioned STIs. Incident cases were assumed to have occurred at the mid-point of the follow-up interval during which the new infection was detected (i.e., “time at risk” for incident cases was represented by the time from baseline to the occurrence of the “first” STI). For the participants who did not contract any incident STIs during the study, the “time at risk” was represented by the time from baseline to the last time they had lab results available. The number of person years at risk was calculated by summing the “time at risk” for each participant. Finally, the incidence density per 100 person years was calculated by taking the ratio between the number of incident cases and number of person years at risk and multiplying it by 100.

Following recommendations for analysis of factorial trials [37], we first assessed whether the interactive formats of each intervention acted independently of each other. For example, for the HIV/STI incidence outcome, we tested whether there was a significant difference –either in direction or magnitude–, with respect to the outcome between the group that received the interactive sex risk intervention (Group C) and the group that received both the interactive sex risk and the interactive injection risk intervention (Group D). For the secondary outcomes, we tested whether an interaction existed between the group that received the interactive injection risk intervention and time, versus the group that received both the interactive sex and interactive injection intervention. For outcomes where there was no evidence of dependency between the two groups in question, the intervention effect was evaluated “at the margins,” whereas for outcomes where such a dependency was present, the intervention effect was evaluated “inside the table.” More specifically, the intervention effect on the primary outcome (i.e., incidence density) was evaluated “at the margins”, whereas the corresponding effect on the secondary outcomes evaluated “inside the table”.

To evaluate the impact of the interactive sexual risk intervention on HIV/STI incidence, we conducted Poisson regression with robust variance estimation via Generalized Estimating Equations (GEE) [38], using the logarithm of the time (years) spent at risk as an offset variable to account for the varying length of time at risk per subject. A natural logarithm link function was used to relate the probabilities of the outcome to the linear combination of the predictors. We first examined the main effects of intervention group (A, B, C, D), study location (Tijuana vs. Ciudad Juarez) and the interaction between the two, respectively. Because the interaction between the intervention group and site was significant, we subsequently performed stratified analyses where the main effect investigated was the effect of the intervention group. GEE with robust variance estimation was chosen as the analytical method for evaluating the primary outcome to correct for over-dispersion. Specifically, one of the main assumptions for the traditional Poisson regression is that the conditional mean equals the conditional variance. At the modeling stage, this assumption was assessed by the deviance statistic divided by the degrees of freedom, which indicated the presence of moderate over-dispersion. In such cases, [38] recommend using robust standard errors for the parameter estimates. The quasi-likelihood under the independence model criterion (QIC) described by Pan [39] was used to compare GEE models to select the most parsimonious model.

To examine our secondary outcomes (frequency of receptive needle sharing, and sharing of injection paraphernalia (cookers, cottons, water and dividing drugs with a used syringe)), we conducted ordinal logistic regression for correlated data via GEE with the correlation matrix estimated empirically from the data. The final analyses for the secondary outcomes were also stratified by site, due to significant differences between sites [29] and significant interactions involving site. Prior to stratification, the main effects investigated were intervention group (Group AC vs. Group BD), visit (Baseline, Visit 2, Visit 3, and Visit 4), study location (Tijuana vs. Ciudad Juarez) and all two-way and three-way interactions between them. After stratification, our primary interest was the interaction between visit and intervention group, with a significant p-value being indicative of an intervention effect. A cumulative logit link function was used to relate the cumulative probabilities of the outcome to the linear combination of the predictors. We chose the ordinal logistic model because our outcomes were ordinal (1 = never, 2 = sometimes, 3 = half the time, 4 = often, 5 = always). The assumption required by the ordinal logistic models is the proportional odds assumption. This assumption was evaluated for all the ordinal logistic regression models by score tests which yielded p-values well above 0.05, suggesting that the ordinal logistic models were reasonable for our data. Additionally, an injection risk index (IRI) score was determined based on an index developed for the Drug User’s Intervention Trial [32], by calculating the average score between responses to injection risk indicators, with higher scores representing higher risk. Gamma regression for correlated data via GEE with an unstructured correlation structure was used to evaluate the impact of the intervention on the mean IRI. A natural logarithm link function was used to relate the mean of the dependent variable to the linear combination of the predictors. The Gamma regression model was chosen primarily because the IRI is a continuous variable with strictly positive values ranging between one and five, with the distribution of values skewed to the right and with the variance of the observations not constant but rather increasing with the mean (as indicated by plotting the residuals from linear normal models against predicted values). Furthermore, the plot of the residuals against of the predicted values from the Gamma models suggested a linear relationship indicating that the gamma models were a reasonable fit for our analyses.

In regression models, we controlled for baseline risk behaviors as covariates. Specifically, at the modeling stage, we considered all potential covariates and their interaction with the main effects and each other. Since interactions usually require more power to detect, a conservative alpha level of 0.10 was used. Graphical displays were used to examine whether the interactions in question were significant and/or confounding. No interactions involving covariates are present in the final models and the covariates included in the final models are all statistically significant at a 0.05 alpha level. Multi-collinearity was assessed for each model and ruled out by the appropriate values of the Variance Inflation Factors and the Condition Indexes. All outcome analyses were based on the assumption that the missing data was missing completely at random (MCAR). This assumption was tested and confirmed by the appropriate values yielded by the Chi-Square test for MCAR [40].

## Results

Of 1132 women who were screened for eligibility, 548 (48.4%) were excluded because they were ineligible (n = 497), or were deemed eligible but did not return for baseline assessment (n = 61). Reasons for exclusion were previously described [29]. Therefore, 584 women enrolled and provided informed consent (284 in Tijuana and 300 in Cd. Juarez), all of whom underwent interviewer-administered surveys and provided biological samples at baseline, and were randomized to Group A (n = 144), Group B (n = 146), Group C (n = 148) and Group D (n = 146).

Over twelve months, only 17 participants (2.9%) did not return for at least one follow-up visit, primarily due to deaths which were unrelated to study participation (n = 10). The remaining 7 participants could not be located. Of the 567 participants who had at least one follow-up visit, an average of 12% per follow-up visit (11% in Tijuana and 13% in Ciudad Juarez) had missing data. However, testing revealed that the missing data was missing completely at random, which allowed for the inclusion of all 567 participants in the outcome analyses.

Compared to participants in Cd. Juarez, Tijuana participants reported higher levels of formal education (8 vs. 6 years, p<.001). In the past month, a higher proportion of Tijuana participants injected drugs at least daily (96.8% vs. 91.3%, p = .005) and often/always injected drugs with a client (47.3% vs. 17.3%, p<.001), compared to participants in Cd. Juarez, but Cd. Juarez participants were more likely to report dividing drugs with a used syringe (74% vs. 63.3%, p = 0.006). Tijuana participants scored higher on the IRI (p = 0.01).

Compared to Tijuana participants, those in Cd. Juarez were younger when they began sex work (median: 19 vs. 20 years, p = 0.02), had more male clients (median: 68 vs. 15 per month, p<0.001), and had more unprotected vaginal/anal sex acts in the past month (median: 33 vs. 25 per month, p<0.001). Although participants from Cd. Juarez earned more money from sex work (median: 1140 vs. 770 USD per month, p<0.001), they earned less per unprotected vaginal sex act (median: 15 vs. 25 USD per act, p<.001). On the other hand, Tijuana participants reported having greater access to condoms (42.2% vs. 17.3%, p<.001) and sterile syringes (43.0% vs. 16.1%, p<.001) in the past year compared to participants in Cd. Juarez. At baseline, Cd. Juarez participants had higher prevalence of lifetime syphilis (i.e., RPR positive and TPPA confirmed syphilis infection; 32.7% vs. 16.2%, p<.001) than participants in Tijuana, but a higher proportion of syphilis cases in Tijuana had titers ≥1∶8 relative to Cd. Juarez (50% vs. 26.8%, p = 0.006).

Comparing baseline characteristics by intervention condition and site suggested that randomization achieved relatively balanced groups (Table 1) with a few exceptions. In Tijuana, participants receiving the interactive formats of both interventions (Group D) were less likely to report dividing drugs with a used syringe at baseline compared to the other groups (p<0.05), and participants receiving the interactive injection risk intervention and didactic sex risk intervention (Group B) were older when they began sex work than participants in Group A (median: 22 years versus 19 years, p<0.05). In Cd. Juarez, participants in Group A were older than those in Group B (p<0.05).

**Table 1:** Descriptive Statistics of Participants by Intervention Group at Baseline, by Study Site (n = 584).

			Tijuana					Ciudad Juarez		
Variable£	Didactic Control(n = 70)	Injection Risk Intervention (n = 71)	Sex Risk Intervention (n = 72)	Both Injection and Sex Risk Intervention (n = 71)	Total (n = 284)	Didactic Control(n = 74)	Injection Risk Intervention (n = 75)	Sex Risk Intervention (n = 76)	Both Injection and Sex Risk Intervention (n = 75)	Total (n = 300)
***Sociodemographics***										
Age (years) ¥¥	31(26,41)	34(28,43)	34(29,41)	33(27,40)	34 (28,41)	35(27,42)	30(24,36)	34(28,38)	34(28,39)	33(27,39)
# of years of education	9(6,11)	8(5,10)	7(6,11)	7(6, 9)	8(6,10)	6(3, 7)	6(4, 8)	6(4, 8)	6(5, 8)	6(4, 8)
Has a spouse or steady partner	28(40.0%)	22(31.0%)	24(33.3%)	33(46.5%)	107(37.7%)	29(39.2%)	32(42.7%)	24(31.6%)	33(44.0%)	118(39.3%)
Earns average of ≥350 USD**	23(32.9%)	23(32.9%)	20(29%)	30(42.3%)	96(34.3%)	40(54.1%)	52(69.3%)	49(64.5%)	39(52.0%)	180(60.0%)
***Drug Use Behaviors***										
Age when first injected drugs (years)	21(18,26)	20(17,24)	19(15.5,23.5)	19(17,23)	20(17,24)	20(17,28)	19(17,24)	20(16,29)	20(18,30)	20(17,27)
Injected > = per day*	69(98.6%)	70(98.6%)	68(94.4%)	68(95.8%)	275(96.8%)	68(91.9%)	70(93.3%)	67(88.2%)	69(92%)	274(91.3%)
Often/always injected drugs with a client**	33(47.1%)	32(45.7%)	32(44.4%)	37(52.1%)	134(47.3%)	13(17.6%)	9(12.0%)	12(15.8%)	18(24.0%)	52(17.3%)
Receptive needle sharing**	69(98.6%)	69(97.2%)	69(95.8%)	64(91.4%)	271(95.8%)	71(95.9%)	71(94.7%)	74(97.4%)	74(98.7%)	290(96.7%)
Divided drugs with used syringe** ¥	46(66.7%)	49(70.0%)	49(69.0%)	34(47.9%)	178(63.3%)	55(74.3%)	62(82.7%)	55(72.4%)	50(66.7%)	222(74.0%)
Used a cooker after someone elsehad used it**	68(98.6%)	69(97.2%)	70(97.2%)	67(95.7%)	274(97.2%)	70(94.6%)	71(94.7%)	74(97.4%)	72(96%)	287(95.7%)
Used a filter after someone else had usedit**	62(88.6%)	65(91.5%)	67(93.1%)	62(88.6%)	256(90.5%)	66(89.2%)	62(82.7%)	63(82.9%)	66(88%)	257(85.7%)
Sharing rinse water**	67(97.1%)	68(95.8%)	67(94.4%)	64(91.4%)	266(94.7%)	70(94.6%)	70(93.3%)	72(94.7%)	71(94.7%)	283(94.3%)
Injection Risk Index Score***	3.6(2.4, 4.2)	3.6(2.4, 4.2)	3.6(2.4, 4.2)	3.6(2.4, 4.2)	3.6(2.4, 4.2)	2.8(2.4, 4.0)	3.2(2.4, 4)	2.8(2.3, 4)	3.4(2.4, 4)	3.2(2.4, 4.0)
***Sex Work Behaviors***										
Age when began to work regularly as aFSW¥	19(16,25)	22(18,27)	19(17,24)	20(18,25)	20(17,25)	19(15,25)	18(16,21)	19(16,25)	19(17,28)	19(16,25)
# Unprotected vaginal/anal sex acts with spouse/steady partner**	8(2,30)	10(2,20)	4(2,36)	10(4,30)	9(2,30)	8(1,30)	13(7,31)	16(7,30)	5(3,23)	10(4,30)
Income earned from sex work (USD)**	660 (250,1500)	660 (240,1430)	820 (340,1390)	900 (420,1650)	770 (305,1500)	1160 (440,2070)	1140 (620,1908)	1200 (645,1818)	975 (450,1900)	1140 (540,1896)
# male clients**	10(6,30)	12(5,30)	16.5(5,30)	20(8,30)	15(6,30)	68(24, 110)	76(36, 100)	66(40, 112)	60(32,96)	68(30, 104)
# vaginal/anal sex acts with clients**	30(12,60)	32(12,59)	36(10,58)	40(15,62)	34(12,60)	90(28, 143)	85(46, 116)	80(48, 130)	75(36, 124)	84(40, 127)
# unprotected vaginal or anal sex actswith clients**	27(7,58)	24(5,48)	27(4,56)	24.5(5,55)	25(5,56)	28(6,69)	34(19,60)	36(14,80)	34(10,60)	33(12,65)
Amount earned per vaginal/anal sex act without condom (USD)	30(20,40)	25(20,40)	28(20,40)	25(20,34)	26(20,40)	15(13,20)	15(10,24)	19(10,29)	20(13,25)	15(10,25)
Arrested in the past 6 months	31(44.3%)	28(40.0%)	27(38.0%)	29(41.4%)	115(40.9%)	37(50.0%)	41(54.7%)	37(48.7%)	32(42.7%)	147(49.0%)
***HIV/STI Services and Lab Test Results***										
Reports more access to condoms*	28(41.2%)	25(38.5%)	31(46.3%)	29(42.6%)	113(42.2%)	10(13.5%)	15(20.0%)	13(17.1%)	14(18.7%)	52(17.3%)
Reports more access to sterile syringes*	26(38.2%)	24(36.4%)	30(44.8%)	36(52.2%)	116(43.0%)	8(10.8%)	12(16%)	12(16%)	16(21.3%)	48(16.1%)
Reports ‘easy access’ to sterile syringes*	57(86.4%)	62(89.9%)	62(91.2%)	57(86.4%)	238(88.5%)	48(65.8%)	55(74.3%)	54(72.0%)	53(70.7%)	210(70.7%)
Tested positive for syphilis	8(11.4%)	16(22.5%)	12(16.7%)	10(14.1%)	46(16.2%)	27(37.0%)	20(27.4%)	25(33.8%)	24(32.4%)	96(32.7%)
Syphilis titers ≥1∶8, among lifetime syphilis cases	4(50.0%)	8(50.0%)	5(41.7%)	6(60.0%)	23(50.0%)	6(22.2%)	8(38.1%)	6(24.0%)	6(25.0%)	26(26.8%)
Tested positive for gonorrhea	3(4.3%)	1(1.4%)	0(0%)	1(1.4%)	5(1.8%)	3(4.1%)	3(4.0%)	1(1.3%)	1(1.3%)	8(2.7%)
Tested positive for Chlamydia	7(10.0%)	10(14.1%)	3(4.2%)	8(11.3%)	28(9.9%)	12(16.2%)	12(16%)	7(9.2%)	11(14.7%)	42(14.0%)
Tested positive for trichomonas	19(27.1%)	24(33.8%)	30(41.7%)	29(40.8%)	102(35.9%)	21(28.4%)	26(34.7%)	27(35.5%)	20(26.7%)	94(31.3%)

* past year;

** past month;

¥ p-value< = 0.05 (Tijuana site);

¥¥ p-value< = 0.05 (Ciudad Juarez Site);

£ The values associated with the continuous variables represent Median (IQR).

*** Comprised of the following: receptive needle sharing, sharing a cooker, cotton filter, or rinse water to prepare drugs for injection after someone else had used it, and using a used syringe to divide drugs. The score was constructed by calculating the average between the responses to these five injection risk indicators (1 = never, 2 = sometimes, 3 = about half the time, 4 = often, and 5 = always), with higher scores representing higher risk.

By definition, all participants reported either receptive needle sharing or injection with used injection equipment within the last month (e.g., receptive needle sharing: 96.2%; sharing a cooker: 96.4%; sharing a cotton filter: 88.0%; sharing rinse water: 94.5%). At baseline, 24.6% of the sample tested positive for syphilis (of whom 34.3% had titers ≥1∶8); 33.6% had trichomoniasis, 12% had Chlamydia and 2.2% had gonorrhea.

In the primary intent-to-treat analysis stratified by site, there was no indication of dependence between the group that received the interactive sexual risk intervention (Group C) and the group that received both the interactive sexual risk and the interactive injection risk intervention (Group D) for Tijuana, suggesting that the intervention effect on HIV/STI incidence could be analyzed “at the margins”. However, we did observe a significant dependence between the two groups for Cd. Juarez, indicating that this analysis should be conducted “inside the table” [37]. In Tijuana, HIV/STI incidence was lower among women randomized to Groups C and D (35.52 and 30.01 per 100 py, respectively) compared to that among women in Group A (64.26 per 100 py) (Table 2). In Cd. Juarez, women randomized to Group C also had lower HIV/STI incidence than women in Group A (34.65 vs. 66.10 per 100 py), but HIV/STI incidence for Group D was not significantly different. The final Poisson regression model for Tijuana (Table 3) found that HIV/STI incidence for women assigned to Group C and D was 62% and 63% lower than women assigned to Group A (i.e., Adjusted relative incidence (ARI: 0.38 and 0.37 (95% CIs: 0.16, 0.89). This model controlled for the number of unprotected sex acts with non-regular clients in the month prior to enrollment, and being arrested in the 6 months prior to enrolment. The final Poisson regression model for Cd. Juarez (Table 4) found that HIV/STI incidence was 56% lower for women randomized to Group C compared to Group A (ARI: 0.44; 95% CI: 0.19, 0.99). This model controlled for the amount earned per unprotected sex act at baseline, and cocaine use in the month prior to baseline.

**Table 2:** HIV/STI incidence density over 12 months: Overall, by intervention group and site.

Group	Intervention group	#of incident cases*	# of peopleat risk		#of py at risk	Incidence densityper 100 py (95% CI)
*Entire Sample*						
**A**	Didactic injection and sex interventions (control)	31	69		47.68	65.02 (42.13,87.91)
**B**	Interactive injection/Didactic sex interventions	31	63		45.82	67.66 (43.84, 91.47)
**C**	Interactive sex/Didactic injection interventions	18	63		51.32	35.08 (18.87,51.28)
**D**	Interactive injection and sex interventions	26	63		48.11	54.04 (33.27, 74.81)
*Tijuana*						
**A**	Didactic injection and sex interventions (control)	18		41	28.01	64.26 (34.58, 93.95)
**B**	Interactive injection/Didactic sex interventions	11		31	23.88	46.067(18.84, 73.29)
**C**	Interactive sex/Didactic injection interventions	9		33	25.34	35.52 (12.31, 58.72)
**D**	Interactive injection and sex interventions	8		32	26.66	30.01 (9.21, 50.81)
*Cd. Juarez*						
**A**	Didactic injection and sex interventions (control)	13		28	19.67	66.10 (30.17,102.02)
**B**	Interactive injection/Didactic sex interventions	20		32	21.94	91.15 (51.20, 131.09)
**C**	Interactive sex/Didactic injection interventions	9		30	25.98	34.65 (12.01, 57.29)
**D**	Interactive injection and sex interventions	18		31	21.45	83.90 (45.14,122.66)

* By STI: 1 HIV, 24 lifetime syphilis, 6 syphilis titers > = 1∶8, 23 Chlamydia, 3 gonorrhea, and 66 trichomoniasis. Fifteen participants presented with more than one STI at the same visit, so incident cases by STI do not add to the same number as the total number of incident HIV/STIs.

**Table 3:** Intervention effects on HIV/STI incidence after 12 months: Tijuana*.

Predictor	Adjusted Relative Risk	95% CI	p-value
Group A: Didactic Sex Risk Intervention+Didactic Injection Risk Intervention			
Group B: Interactive Injection Risk and Didactic Sex Risk Intervention	0.88	0.40, 1.94	0.74
Group C: Interactive Sex Risk Intervention and Didactic Injection Risk Intervention	0.38	0.16, 0.89	0.03
Group D: Interactive Sex Risk Intervention+Active Injection Risk Intervention	0.37	0.16, 0.89	0.03
# of unprotected sex acts with non-regular clients for month prior to enrollment	1.01	1.01, 1.02	<0.001
Arrested during the six months prior to enrollment	2.68	1.39, 5.15	0.003

* excluding women who tested HIV-positive or had STIs at baseline.

**Table 4:** Intervention effect on 12 months HIV/any STI incidence rate: Ciudad Juarez*.

Predictor	Adjusted Relative Risk	95% CI	p-value
Intervention Group (ref = Didactic)			
Interactive Injection Risk Intervention	1.15	0.58, 2.28	0.68
Interactive Sex Risk Intervention	0.44	0.19, 0.99	0.05
Interactive Sex Risk Intervention & Interactive Injection Risk	1.12	0.56, 2.25	0.76
Amount earned per unprotected sex act at baseline (per USD increase)	1.02	1.00, 1.05	0.04
Used cocaine the month prior to baseline	1.66	0.98, 2.80	0.05

* excluding women who tested HIV-positive or had STIs at baseline.

**Figure 1:**
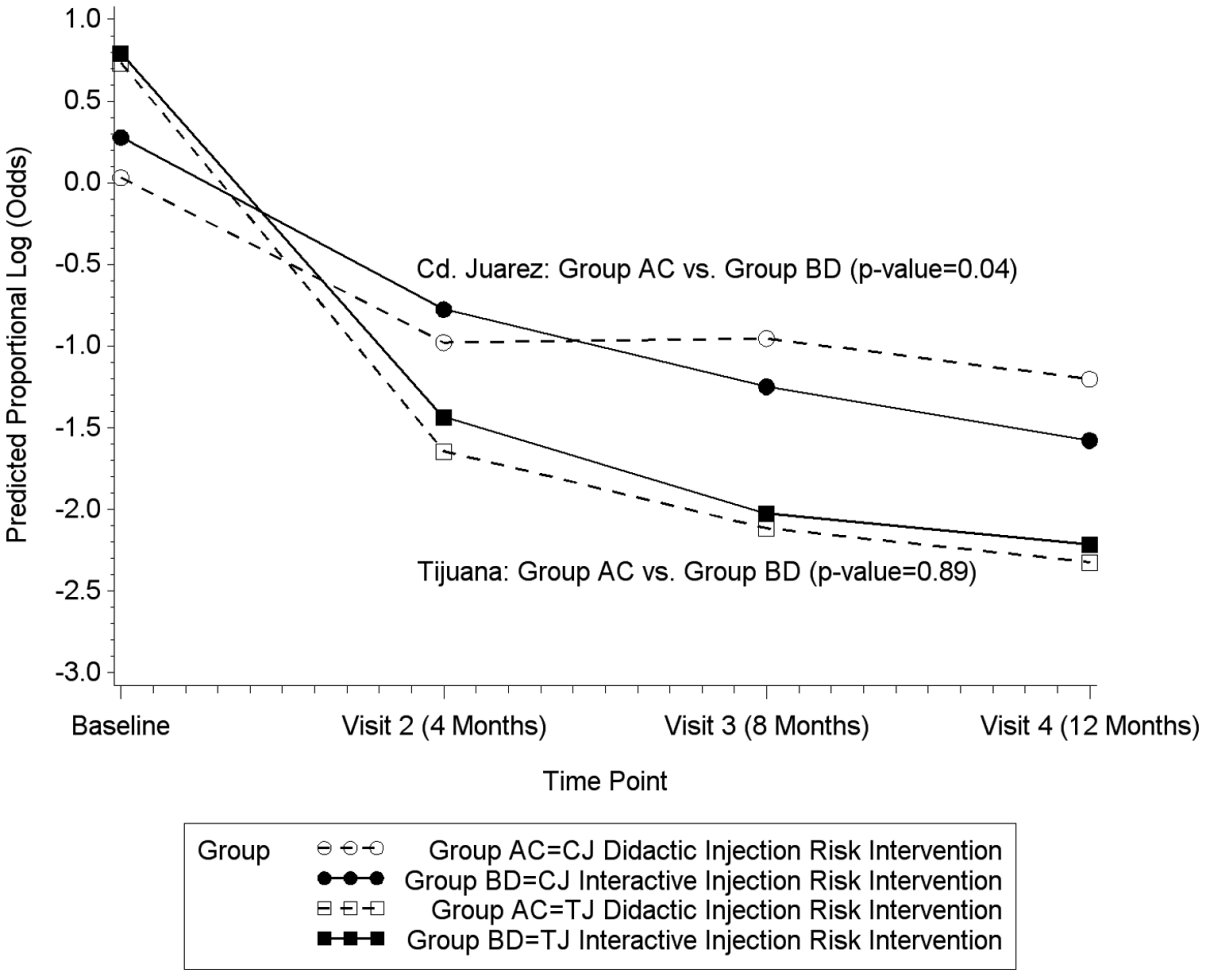
Changes in Proportional Log Odds of Receptive Needle Sharing among Participants in Tijuana and Ciudad Juarez Receiving Interactive or Didactic Injection Risk Interventions.

The analysis of secondary outcomes revealed no significant dependence between the group that received the interactive injection risk intervention (Group B) and the group that received both the active injection and the active sex intervention (Group D) at any of the time points, indicating that these analyses could be conducted “at the margins” for both sites. For Cd. Juarez, the interactive injection risk intervention was associated with significant declines in receptive needle sharing (Figure 1; p = 0.04), and IRI score (p = 0.01; results not shown) and sharing of individual injection paraphernalia items (i.e., cookers, filters, rinse water; results not shown). In an ordinal regression model, the interaction between visit 4 and intervention group was significant (p<0.0001), indicating that a significantly higher reduction in the proportional odds of receptive needle sharing was achieved in the intervention group as compared to the control group after 12 months of follow-up (results not shown).

Although the slope of the decline in receptive needle sharing and IRI score was steeper for Tijuana compared to Cd. Juarez, there were no significant differences in the proportional odds of receptive needle sharing (Figure 1) or the predicted mean IRI scores comparing groups in Tijuana that received the interactive injection risk intervention to those that did not (results not shown).

## Discussion

This combination prevention trial conducted in two Mexican-U.S. border cities is the first to achieve simultaneous, significant reductions in both sexual and injection risk behaviors among sex workers who inject drugs. Analyses of the interactive safer sex intervention extend the findings of the earlier *Mujer Segura* trial [26], and confirm that FSW-IDUs can experience significant reductions in HIV/STI incidence in the context of their drug use and/or that of their commercial sexual partners, as other researchers have advocated [41]. Indeed, the magnitude of the risk reduction in this trial (50–60%) surpassed that of the *Mujer Segura* trial, and the strong intervention effect we observed was sustained over one year in both cities. The impact of the intervention on HIV/STI incidence was slightly greater in Tijuana than Cd. Juarez, perhaps because women in the former city reported having greater access to condoms.

Our findings also demonstrate that condom provision and HIV prevention education delivered in a lecture format is not sufficient for achieving reductions in HIV/STI incidence among FSW-IDUs. In the case of male condoms, negotiation skills are typically required to ensure that sex workers can convince their partners to use them. Our findings are consistent with other interventions that have shown that FSWs can be taught to successfully negotiate condom use with clients in other settings[42]–[45], but extends this research since our intervention was associated with significant reductions in HIV/STI incidence and can be delivered within 30 minutes by peer workers with minimal training. An intervention is underway to determine whether a similar intervention approach is efficacious among male clients of FSWs in Tijuana.

Although the two groups of women who received the interactive sexual risk intervention in Tijuana experienced nearly identical reductions in HIV/STI incidence, only the group receiving the interactive sexual risk intervention in combination with the didactic format of the injection risk intervention achieved a significant decrease in HIV/STI incidence. Women in Cd. Juarez receiving the interactive formats of both interventions may not have experienced similar declines in HIV/STI incidence because they tended to engage in higher risk sexual behaviors than their counterparts in Tijuana. A more intensive intervention may be needed for the highest risk subgroup, among which simultaneous risk reductions in both injection and sexual risks may be more challenging with brief interventions.

**Figure 2:**
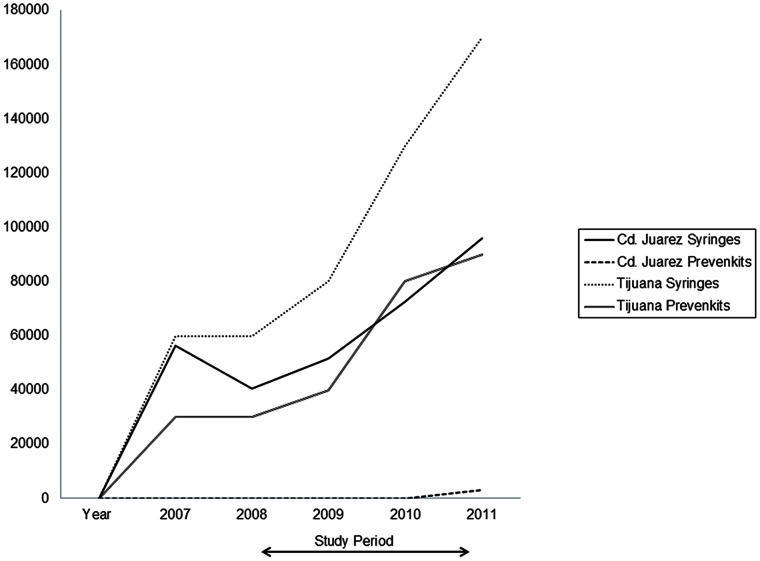
Reported Number of Syringes Exchanged and Prevenkits* Distributed in Tijuana and Ciudad Juarez: 2007–2011. *Safer injection kits including sterile cotton, cooker and water.

Interestingly, the interactive injection risk intervention was associated with significant reductions in receptive needle sharing and sharing of paraphernalia in Cd. Juarez, but not in Tijuana. In Tijuana, women randomized to both the interactive and didactic formats of the intervention dramatically reduced their injection risk behaviors relative to baseline–and to a greater extent than women in Cd. Juarez– but there was no difference between groups of Tijuana women who received the two intervention formats. This was an unexpected finding, but we were aware that the number of syringes and kits containing injection paraphernalia (‘prevenkits’) increased markedly in Tijuana versus Cd. Juarez during the study period (Figure 2). In a post-hoc analysis, we tested the hypothesis that increased access to syringes may have dampened the intervention effect. Specifically, we offered the variable ‘reported easy access to sterile syringes’ into the ordinal regression model for Cd. Juarez and found that the syringe access variable became significant (POR: 0.69; 95% CI: 0.50–0.94) and the variable representing the interactive injection risk intervention lost statistical significance (POR = 1.06; 95% CI: 0.79–1.42), in support of our hypothesis. These findings suggest that the didactic format of the injection risk intervention coupled with expanded access to sterile injection equipment is both necessary *and* sufficient for achieving significant reductions in injection risk, supporting literature that underscores the critical role of sterile syringe coverage through syringe exchange programs (NSPs), pharmacies and over-the-counter syringe sales [46], [47].

NSPs now exist in six Mexican states including Baja California and Chihuahua; however, syringe coverage is uneven and very low. Although it is legal to purchase syringes in Mexico without a prescription, IDUs report persistent barriers to purchasing and carrying syringes in both Tijuana and Cd. Juarez [48], [49]. Being arrested for carrying used or unused syringes by police has been associated with both receptive needle sharing [49], and HIV infection [5] in both cities. This suggests that structural interventions such as police education programs will be needed to ensure that behavior changes achieved by this and other interventions are not erased.

Our findings from Cd. Juarez suggest that the interactive format of the injection risk intervention has value in settings with sub-optimal syringe access, such as those with limited resources or laws and policies that restrict IDUs’ access to syringes. Despite a plethora of studies demonstrating the efficacy of NSPs for reducing HIV transmission, they exist in only 82 countries where HIV infection has been reported among IDUs, and coverage is extremely low at an estimated 22 syringes distributed per IDU per year [50] which is insufficient to prevent HIV transmission. In most countries, no access to syringes is provided in jails/prisons, despite ample access to drugs, suggesting that our injection risk intervention may benefit IDUs in detention facilities.

Our study was limited by the fact that it was not originally designed to test the efficacy of these interventions by site. We were also under-powered to examine the impact of the sexual risk intervention on incidence of HIV or individual STIs alone; indeed, there was only one incident HIV case during follow-up. Further, our study focused on condom negotiation within the context of sexual transactions, rather than intimate relationships with non-paying partners. Despite these shortcomings, we observed significant intervention effects on combined HIV/STI incidence that did not wane after 12 months. Our analysis of injection risk outcomes was based on self-reported behaviors, rather than a biologic outcome because HCV prevalence exceeded 90% among IDUs in both cities [51]. While reports of injection behaviors could have been subject to socially desirable responding, we had no reason to expect that this would occur differentially across intervention conditions, because all four groups received the same interventions and only the format of the interventions differed. Further, since interviewers were blind to intervention group, there is no reason to expect that information elicited from participants would differ across intervention groups.

In summary, we found that a brief, interactive counseling session was successful in reducing HIV/STI incidence by over 50% over a twelve month follow-up period among FSW-IDUs in two Mexican-U.S. border cities. With respect to reducing injection risk behaviors, our results indicate that it is more important to scale-up free access to injection equipment at the community-level with minimal harm reduction education than to provide an intensive individual-level intervention without adequate syringe coverage. However, our interactive injection risk intervention may be useful for many settings where sterile syringe coverage cannot be sufficiently increased. Given that both interventions are brief and can be offered with minimal training, future studies are needed to examine their contribution to combination prevention approaches in settings where FSWs and IDUs receive other services such as drug abuse treatment programs, NSPs, reproductive health clinics and detention facilities, as well as structural interventions such as those involving managers, madams and clients.

## Supporting Information

Checklist S1 **CONSORT Checklist.**
(DOC)

Protocol S1 **Trial Protocol.**
(PDF)
